# Trust and anxiety as primary drivers of digital health acceptance in multiple sclerosis: toward an extended disease-specific technology acceptance model

**DOI:** 10.3389/fdgth.2026.1763329

**Published:** 2026-03-13

**Authors:** Felix Höpfl, Mira Brundiers

**Affiliations:** Faculty of Applied Health and Social Sciences, Rosenheim Technical University of Applied Sciences, Rosenheim, Germany

**Keywords:** multiple sclerosis, digital health, artificial intelligence, technology acceptance, trust in technology, technological anxiety, wearables

## Abstract

**Background:**

Digital health applications and AI-supported wearables may benefit people with Multiple Sclerosis (MS), yet fluctuating cognitive and physical symptoms could shape adoption in ways not fully captured by traditional acceptance models.

**Objective:**

To identify determinants of digital health acceptance in MS, focusing on emotional factors and disease-related moderators, and to compare these patterns with individuals living with other chronic conditions.

**Methods:**

An online survey (Winter 2024/2025) assessed Technology Acceptance Model (TAM) and Unified Theory of Acceptance and Use of Technology (UTAUT) constructs in MS patients (*n* = 64) and a comparison group (*n* = 14). Measures included Perceived Usefulness (PU), Perceived Ease of Use (PEOU), Behavioral Intention (BI), Social Influence (SI), Trust in Technology (TT), Technological Anxiety (TA), and self-reported wearable/app use.

**Results:**

Groups did not differ significantly in PU, PEOU, BI, or SI (all *p* > .05), though between-group comparisons should be interpreted cautiously given the small comparison group size (*n* = 14). However, MS participants reported substantially lower regular wearable use [*χ*^2^(2) = 7.83, *p* = .020]. TT (*β* = .52, *p* < .001) and TA (*β* = –.38, *p* < .001) were the strongest predictors of BI, whereas PU and PEOU contributed minimally. Symptom severity moderated acceptance pathways, weakening PEOU effects and amplifying TA effects.

**Conclusion:**

Findings reveal an intention–behavior gap in MS and show that emotional and capability-related factors outweigh cognitive predictors. We outline foundational elements of an Extended Disease-Specific Technology Acceptance Model for MS integrating trust, anxiety, and symptom burden. Digital health tools for MS should prioritize trust-building and anxiety-reducing design features.

## 1 Introduction

Artificial intelligence (AI) and digital health applications are increasingly integrated into chronic disease management. In Multiple Sclerosis (MS), characterized by fluctuating symptoms including fatigue, heat sensitivity, gait instability, and cognitive impairment (1), continuous monitoring through wearables and AI-enhanced applications offers opportunities for symptom detection, personalized alerts, and adaptive interventions (2–4).

Despite potential advantages, adoption rates among MS patients remain uneven. Prior work indicates that MS patients often express positive attitudes toward digital health tools but show comparatively low usage patterns (5). This discrepancy is particularly concerning given MS disease burden, including elevated mortality risk and comorbidity rates (6), alongside cognitive impairments affecting daily functioning (1). Traditional acceptance models may not adequately capture these MS-specific adoption barriers.

The Technology Acceptance Model (TAM) (7) and the Unified Theory of Acceptance and Use of Technology (UTAUT) (8, 9) are foundational frameworks for predicting digital health adoption. TAM emphasizes Perceived Usefulness (PU) and Perceived Ease of Use (PEOU) as key determinants, while UTAUT incorporates social and contextual factors.

These models demonstrate robust predictive validity across healthcare technologies (10–12), though extensions have incorporated additional constructs for vulnerable populations (13, 14).

However, emotional factors such as Trust in Technology (TT) and Technological Anxiety (TA) have gained prominence in digital health research, particularly for vulnerable populations (15–17). Trust and anxiety emerge as critical determinants in healthcare technology adoption (18–20), often outweighing traditional TAM constructs among chronic disease patients (21). These constructs may be especially relevant for MS due to disease uncertainty, cognitive demands (1), and symptom unpredictability (3). Privacy concerns represent additional trust-related barriers in healthcare contexts (22). Furthermore, existing models do not account for disease-specific moderators such as symptom severity, which may fundamentally alter acceptance pathways.

Traditional acceptance models assume behavioral intention directly predicts actual use. Yet meta-analytic evidence reveals substantial intention-behavior gaps, particularly pronounced in conditions involving cognitive impairment (23, 24). For MS specifically, cognitive deficits in information processing and executive function (1) may disrupt consistent technology interaction independent of perceived usefulness or ease of use.

We introduce an exploratory MS-specific extension of the Technology Acceptance Model (D-TAM). The purpose is not to propose a full structural model, but to identify which emotional and disease-specific factors appear to meaningfully shape acceptance patterns.

### 1.1 Study objectives

This exploratory study aims to:

1. Compare acceptance factors between MS patients and individuals with other chronic conditions
2. Analyze predictors of behavioral intention and actual technology usage
3. Investigate moderating effects of MS symptom severity on acceptance pathways
4. Develop foundational elements toward a disease-specific extension of TAM/UTAUT for MS populations

## 2 Methods

### 2.1 Study design and recruitment

This cross-sectional study was conducted via online survey using the TIVIAN platform during December 2024 and January 2025. Participants were recruited through patient advocacy networks, with substantial support from the German MS Society (Deutsche Multiple Sklerose Gesellschaft—DMSG), which facilitated access to individuals with MS diagnosis. Additional participants with other chronic conditions were recruited through chronic disease forums and social media health communities.

### 2.2 Participants

The survey yielded *N* = 157 responses. After excluding incomplete responses (dispcode ≠ 31, *n* = 37) and individuals without chronic illness (*n* = 75), the final analytical sample comprised *N* = 78 participants:

- MS group: *n* = 64 (82.1%)
- Other chronic conditions: *n* = 14 (17.9%)
  - Conditions included: diabetes (*n* = 4), asthma (*n* = 3), chronic inflammatory bowel disease (*n* = 3), cardiovascular disease (*n* = 2), cancer (*n* = 2)

### 2.3 Ethical considerations

The study was designed according to ethical principles for human research. A formal ethics self-assessment was conducted following German Association for Health Economics and Outcomes Research (GEHBA) guidelines. Based on this assessment, the study was classified as minimal risk and did not require institutional ethics committee approval.

While persons with Multiple Sclerosis represent a vulnerable population due to potential cognitive impairment, physical limitations, and disease-related psychosocial stress, the minimal risk classification remained appropriate because: (a) no clinical intervention or manipulation was involved, (b) anonymous data collection with no identifiable health information, (c) cognitive demands were minimized through clear, jargon-free language and optional breaks, (d) voluntary participation with explicit withdrawal rights, and (e) no sensitive clinical data beyond general symptom severity ratings were collected.

All participants provided digital informed consent before participation. The consent form explained study purposes, data handling procedures, voluntary participation, and withdrawal rights. Data were collected and stored in compliance with the European General Data Protection Regulation (GDPR). No personally identifiable information was collected, and IP addresses were not recorded.

### 2.4 Measures

#### 2.4.1 Technology acceptance constructs

Six core acceptance constructs were assessed using 5-point Likert scales (1 = strongly disagree, 5 = strongly agree), with five items per construct adapted from validated TAM/UTAUT scales:

1. Perceived Usefulness (PU): Extent to which users believe technology would improve health management
2. Perceived Ease of Use (PEOU): Perceived effort required to use the technology
3. Behavioral Intention (BI): Willingness to use or continue using the technology
4. Social Influence (SI): Perceived social pressure or encouragement from important others
5. Trust in Technology (TT): Confidence in accuracy, reliability, and data security
6. Technological Anxiety (TA): Apprehension or fear regarding technology use

Items were contextualized with MS-relevant examples [e.g., “An AI app that warns me when my body temperature rises too much would help me better manage my MS symptoms” (PU); “I trust that AI-based health apps analyze my health data accurately” (TT)]. Negatively worded items were reverse-coded.

#### 2.4.2 Actual technology Use

Actual behavior was measured using two items:

- Wearable use (v_42): “Do you currently use a wearable device (e.g., smartwatch, fitness tracker) to monitor health data?” (0 = no, 1 = occasionally, 2 = regularly)
- AI health app use (v_12): “Do you currently use AI-supported health apps?” (0 = no, 1 = occasionally, 2 = regularly)

#### 2.4.3 MS symptom severity

MS participants rated current severity of three core symptom domains on 5-point scales:

- Fatigue/exhaustion
- Cognitive challenges (concentration, memory)
- Motoric impairments (coordination, gait)

A composite symptom severity index was computed as the mean of these three ratings (*α* = .76).

### 2.5 Statistical analysis

Analyses were conducted using SPSS 28.0 and included:

1. Reliability analysis: Cronbach's *α* for internal consistency
2. Exploratory Factor Analysis (EFA): Principal axis factoring with oblimin rotation to examine construct distinctiveness in MS populations. While the scales were adapted from validated TAM/UTAUT instruments, EFA was conducted to verify that these constructs remain distinct in MS samples, given potential cognitive overlap due to disease-related factors.
3. Group comparisons: Given the small comparison group (*n* = 14), effect sizes were assessed using Cliff's Delta (δ) (25), a non-parametric measure robust to small samples and non-normal distributions. Cliff's Delta ranges from −1 to +1, with values interpreted as: negligible (|*δ*| < .15), small (.15 ≤ |*δ*| < .33), medium (.33 ≤ |*δ*| < .47), and large (|*δ*| ≥ .47). Independent samples t-tests were conducted for descriptive purposes. Chi-square tests were used for categorical usage variables.
4. Regression analyses: Multiple linear regression predicting BI; ordinal logistic regression for usage outcomes
5. Moderation analyses: Hierarchical regression testing symptom severity ×  acceptance construct interactions, with continuous interaction terms and mean-centered predictors (26).
6. Statistical significance was set at *α* = .05 (two-tailed). Given the exploratory nature of moderation analyses and the small comparison group, effect sizes were prioritized over *p*-values for between-group comparisons.

## 3 Results

### 3.1 Sample characteristics

The MS group (*n* = 64) had a mean age of 46.8 years (SD = 11.2) and was predominantly female (73.4%), with average disease duration of 8.4 years (SD = 6.1) and moderate symptom severity (M = 3.2, SD = 0.9). The comparison group (*n* = 14) showed similar demographic profiles: mean age 48.3 years (SD = 9.7), 71.4% female, average disease duration 7.2 years (SD = 5.8). No significant differences emerged in age [t(76) = 0.52, *p* = .61], gender distribution (*χ*^2^ = 0.03, *p* = .87), or disease duration [t(76) = 0.71, *p* = .48], supporting comparability of groups on key demographic variables, though the small comparison group size (*n* = 14) limits statistical power for detecting subtle differences.

### 3.2 Reliability and construct validity

All scales demonstrated good to excellent internal consistency (Table 1):

- Perceived Usefulness: *α* = .89
- Perceived Ease of Use: *α* = .87
- Behavioral Intention: *α* = .91
- Social Influence: *α* = .83
- Trust in Technology: *α* = .88
- Technological Anxiety: *α* = .85

**Table 1:** Descriptive statistics, reliability coefficients (Cronbach's α), and effect size comparisons between MS and other chronic condition groups.

Construct	Total (*N* = 78)		MS (*n* = 64)	Other (*n* = 14)	Group comparison			
	M (SD)	*α*	M (SD)	M (SD)	Cliff's *δ*	Interp.	t	*p*
PU	3.88 (0.73)	.89	3.87 (0.74)	3.91 (0.68)	+0.02	Negligible	0.21	.84
PEOU	3.57 (0.80)	.87	3.54 (0.82)	3.68 (0.71)	−0.09	Negligible	0.62	.54
BI	3.74 (0.87)	.91	3.72 (0.89)	3.85 (0.76)	−0.02	Negligible	0.55	.58
SI	3.24 (0.93)	.83	3.21 (0.94)	3.35 (0.88)	+0.14	Negligible	0.54	.59
TT	3.42 (0.90)	.88	3.38 (0.91)	3.67 (0.84)	+0.11	Negligible	1.15	.25
TA	2.78 (1.01)	.85	2.84 (1.02)	2.53 (0.96)	+0.08	Negligible	1.11	.27

Note: PU = perceived usefulness; PEOU = perceived ease of use; BI = behavioral intention; SI = social influence; TT = trust in technology; TA = technological anxiety. α = Cronbach's alpha. δ = Cliff's Delta (negligible: |δ| < .15; small: .15–.32; medium: .33–.46; large: ≥ .47). Positive δ values indicate higher scores in the MS group. All scales ranged from 1 to 5. Between-group comparisons should be interpreted cautiously due to the small comparison group (*n* = 14).

Exploratory factor analysis (KMO =.84, Bartlett's test: *χ*^2^ = 892.4, *p* < .001) supported a five-factor solution explaining 72.3% of variance. TT and TA emerged as distinct, robust factors. PU and PEOU showed partial overlap in the MS subsample (factor correlation *r* = .58), suggesting MS patients may perceive these constructs as less differentiated than traditional models assume—possibly reflecting cognitive demands that make usefulness and ease of use feel interconnected.

Bivariate intercorrelations among all acceptance constructs are presented in Table 2.

**Table 2:** Intercorrelations (total sample, *N* = 78).

Variable	1	2	3	4	5	6
1. PU	—					
2. PEOU	.51**	—				
3. BI	.49**	.44**	—			
4. SI	.38**	.32**	.41**	—		
5. TT	.45**	.48**	.67**	.39**	—	
6. TA	−.31**	−.39**	−.54**	−.28*	−.58**	—

Note: 1 = PU (Perceived Usefulness); 2 = PEOU (Perceived Ease of Use); 3 = BI (Behavioral Intention); 4 = SI (Social Influence); 5 = TT (Trust in Technology); 6 = TA (Technological Anxiety). **p < .01; *p < .05.

Given the sample size (*n* = 78), the exploratory factor analysis should be interpreted with caution. Recommended subject-to-item ratios were not fully met, and the analysis was therefore used only to examine whether the adapted constructs showed the expected approximate simple structure in an MS population. Reliability indices (*α* = .78–.91) were used as the primary indicators of construct quality, and the EFA results are presented as supportive, not confirmatory, evidence of construct separability.

The complete item list and corresponding factor loadings for all constructs are provided in Supplementary Table S1.

### 3.3 MS vs. other chronic conditions

#### 3.3.1 Acceptance constructs

Effect size analyses using Cliff's Delta revealed negligible differences between groups on all core acceptance constructs (Table 1):

- Perceived Usefulness: MS (M = 3.87, SD = 0.74) vs. Other (M = 3.91, SD = 0.68), *δ* =  + 0.02 (negligible)
- Perceived Ease of Use: MS (M = 3.54, SD = 0.82) vs. Other (M = 3.68, SD = 0.71), *δ* = −0.09 (negligible)
- Behavioral Intention: MS (M = 3.72, SD = 0.89) vs. Other (M = 3.85, SD = 0.76), *δ* = −0.02 (negligible)
- Social Influence: MS (M = 3.21, SD = 0.94) vs. Other (M = 3.35, SD = 0.88), *δ* =  + 0.14 (negligible, approaching small)
- Trust in Technology: MS (M = 3.38, SD = 0.91) vs. Other (M = 3.67, SD = 0.84), *δ* =  + 0.11 (negligible)
- Technological Anxiety: MS (M = 2.84, SD = 1.02) vs. Other (M = 2.53, SD = 0.96), *δ* =  + 0.08 (negligible)

All effect sizes fell below the threshold for small effects (|*δ*| < .15), suggesting that MS and other chronically ill individuals may hold comparable attitudes toward digital health technologies, though the small comparison group (*n* = 14) limits definitive conclusions. Independent samples t-tests confirmed the absence of significant differences (all *p* > .05), but these null findings should be interpreted with caution given limited statistical power.

#### 3.3.2 Actual technology use

Despite comparable intentions, MS patients showed significantly lower actual wearable use:

- Wearable use: *χ*^2^(2) = 7.83, *p* = .020
  - MS: 34.4% regular, 31.3% occasional, 34.4% non-users
  - Other: 57.1% regular, 35.7% occasional, 7.1% non-users

AI health app use showed a similar pattern, though not reaching statistical significance:

- AI app use: *χ*^2^(2) = 5.06, *p* = .080
  - MS: 18.8% regular, 28.1% occasional, 53.1% non-users
  - Other: 35.7% regular, 35.7% occasional, 28.6% non-users

### 3.4 Predictors of behavioral intention

Multiple regression analysis (Table 3) revealed that emotional factors dominated cognitive constructs in predicting BI [*R*^2^ = .61, F(5,72) = 22.4, *p* < .001]:

**Table 3:** Multiple linear regression predicting Behavioral Intention (BI).

Predictor	B	SE	*β*	t	*p*	95% CI (Lower)	95% CI (Upper)
PU	0.21	0.12	.18	1.76	.081	−0.03	0.45
PEOU	0.15	0.10	.14	1.55	.123	−0.05	0.35
SI	0.22	0.09	.23	2.35	.021	0.04	0.40
TT	0.50	0.10	.52	5.14	<.001	0.30	0.70
TA	−0.33	0.08	−.38	−4.21	<.001	−0.49	−0.17

Note: β = standardized regression coefficient. R² = .61. *N* = 78.

Significant predictors:

- Trust in Technology: *β* = .52, *p* < .001
- Technological Anxiety: *β* = -.38, *p* < .001
- Social Influence: *β* = .23, *p* = .021

Non-significant predictors:

- Perceived Usefulness: *β* = .18, *p* = .081
- Perceived Ease of Use: *β* = .14, *p* = .123

When TT and TA were excluded from the model, PU became significant (*β* = .41, *p* = .002), suggesting emotional factors may suppress or suppress the influence of cognitive appraisal in MS contexts.

### 3.5 Predictors of actual use

Ordinal logistic regression predicting wearable use frequency revealed:

- Behavioral Intention: OR = 2.14, *p* = .012
- Trust in Technology: OR = 1.87, *p* = .039
- Symptom Severity: OR = 0.61, *p* = .028 (higher severity → lower use)

The intention-behavior correlation was moderate (*r* = .44, *p* < .001), confirming the gap between expressed willingness and actual adoption.

### 3.6 Moderation by symptom severity

Hierarchical regression tested interactions between symptom severity and acceptance constructs (Table 4). In the MS subsample:

**Table 4:** Hierarchical regression analysis testing moderation effects of symptom severity on acceptance pathways (MS subsample).

Interaction Term	*β*	t	*p*	*ΔR* ^2^
Symptom Severity × PU → BI	−.12	−1.18	.24	.01
Symptom Severity × PEOU → BI	−.24	−2.08	.042	.05
Symptom Severity × TT → BI	−.09	−0.73	.47	.01
Symptom Severity × TA → BI	−.28	−2.41	.019	.06

Note: Interaction terms were computed using mean-centered predictors. β = standardized regression coefficient.

Significant interactions:

- Symptom Severity × PEOU → BI: *β* = −.24, *p* = .042
  - At low symptom severity: PEOU → BI (*β* = .32, *p* = .018)
  - At high symptom severity: PEOU → BI (*β* = .08, *p* = .56)
- Symptom Severity × TA → BI: *β* = −.28, *p* = .019
  - At low symptom severity: TA → BI (*β* = −.21, *p* = .12)
  - At high symptom severity: TA → BI (*β* = −.49, *p* = .003)

Non-significant interaction:

- Symptom Severity × TT → BI: *β* = −.09, *p* = .47 (trust effects remained stable)

These findings indicate that symptom burden fundamentally alters acceptance pathways: ease of use becomes less relevant under high symptom load, while anxiety becomes disproportionately influential.

## 4 Discussion

The present study was designed as an exploratory investigation into determinants of digital health acceptance among persons with Multiple Sclerosis (MS), with the aim of identifying disease-specific patterns that may not be adequately captured by established technology acceptance models developed in predominantly healthy populations. Rather than testing a predefined structural model, the analyses sought to generate hypotheses and inform conceptual extensions by examining how emotional factors, symptom severity, and classical acceptance constructs interact within an MS context.

**Figure 1:**
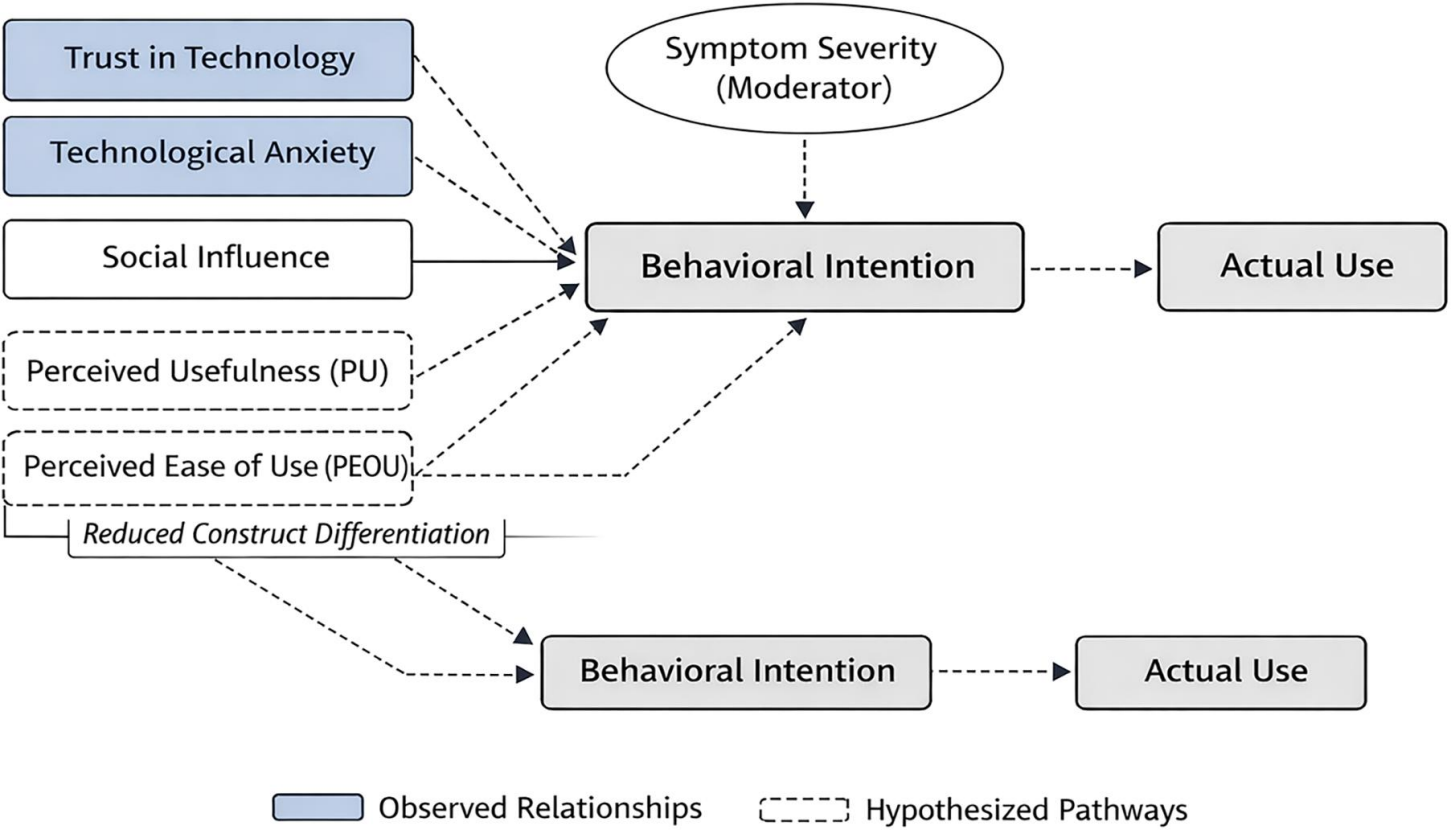
Conceptual extension of technology acceptance for multiple sclerosis (exploratory).

Across the analyses, several systematic patterns emerged that warrant careful interpretation. In particular, emotional determinants—most notably trust in technology and technological anxiety—were consistently associated with behavioral intention, while traditional cognitive evaluations such as perceived usefulness and perceived ease of use appeared attenuated and partially overlapping. Moreover, symptom severity emerged as a contextual factor that reshaped acceptance pathways and weakened the translation of intention into actual use. Taken together, these findings suggest that technology acceptance in MS is characterized less by stable cognitive appraisals and more by fluctuating emotional and capability-related constraints, providing a basis for the conceptual framework outlined in Figure 1.

In this context, the attenuated association between behavioral intention and actual use observed in the MS group warrants further consideration. Meta-analytic evidence from health psychology demonstrates that behavioral intention typically explains only a limited proportion of behavioral variance, accounting for approximately 20%–30% across health-related behaviors (24). Importantly, the intention–behavior gap has been shown to widen under conditions of increased cognitive or self-regulatory burden, including impaired executive functioning and reduced action control (23). Against this backdrop, the present findings may indicate that neurological symptom fluctuations and cognitive fatigue in MS further constrain the translation of intention into sustained technology use, rather than constituting a fundamentally different acceptance mechanism.

### 4.1 Understanding the intention-behavior gap in MS

While traditional models assume behavioral intention directly predicts technology use (7, 8), our findings indicate this pathway is substantially weaker in MS. Despite comparable intentions (though comparability should be interpreted cautiously given *n* = 14 for the comparison group), MS patients were 40% less likely to use wearables regularly [34.4% vs. 57.1%, *χ*^2^(2) = 7.83, *p* = .020]. This gap reflects disease-specific capability constraints—fatigue, symptom unpredictability, and cognitive load—that disrupt consistent technology interaction even when attitudes are positive (6). Standard acceptance models, developed for healthy populations, do not account for such fluctuating capabilities, highlighting the need for frameworks incorporating physical and cognitive moderators.

### 4.2 Emotional factors dominate cognitive appraisal under MS conditions

Across analyses, emotional determinants emerged as the most robust predictors of behavioral intention among participants with Multiple Sclerosis. Trust in Technology and Technological Anxiety showed substantially stronger associations with intention than classical cognitive acceptance constructs such as perceived usefulness and perceived ease of use. While traditional Technology Acceptance Model formulations emphasize cognitive evaluation as the primary driver of adoption, the present findings suggest that under MS-specific conditions, emotional responses play a more prominent and immediate role in shaping acceptance decisions.

Importantly, the dominance of emotional factors does not appear to result from elevated absolute levels of trust or anxiety in the MS group. Group comparisons revealed negligible differences between MS participants and individuals with other chronic conditions on both constructs, though these comparisons are exploratory given the small comparison group (*n* = 14) and should be interpreted with caution. Instead, moderation analyses indicate that identical emotional states exert differential behavioral influence depending on symptom burden, with anxiety becoming more consequential and ease-of-use considerations diminishing as symptom severity increases. This pattern suggests that emotional determinants may function as sensitivity amplifiers under conditions of fluctuating cognitive and physical capacity.

A theoretically informative finding in this context is the partial overlap between perceived usefulness and perceived ease of use observed within the MS sample. Although conceptually distinct in classical acceptance models, these constructs appeared less differentiated, suggesting that users with MS may evaluate usefulness and usability in a more integrated manner. One plausible interpretation is that cognitive load and fatigue reduce evaluative differentiation, leading users to rely on more global, effort-based judgments rather than analytically separating functional benefit from usability characteristics. From this perspective, a system that is experienced as difficult to use may also be perceived as less useful—not due to its objective functionality, but because the effort required to engage with the technology constrains its perceived value.

This interpretation aligns with broader cognitive load and self-regulation frameworks, which posit that under reduced cognitive capacity, evaluative judgments become less granular and more strongly influenced by perceived effort and emotional response, a pattern also observed in digital health contexts characterized by limited digital health literacy and resource constraints (27, 28). In the context of MS, where fatigue, slowed information processing, and symptom unpredictability are common, such effort-based integration of cognitive appraisals may represent an adaptive response to fluctuating capability rather than a deficit in judgment. Consequently, the relative explanatory power of perceived usefulness and ease of use may be state-dependent, diminishing during periods of higher symptom burden and becoming more salient when cognitive and physical resources are more stable.

Taken together, these findings suggest that emotional determinants and capability-related constraints jointly reshape acceptance processes in MS. Rather than replacing cognitive appraisal, emotional factors appear to restructure the conditions under which cognitive evaluations remain influential, setting the stage for the moderating role of symptom severity examined in the following section.

### 4.3 Symptom severity reshapes acceptance pathways

The present findings indicate that symptom severity plays a contextual and moderating role in shaping technology acceptance among persons with Multiple Sclerosis. Rather than acting as an independent determinant of behavioral intention, symptom severity appears to alter the relative influence of emotional and cognitive acceptance constructs, thereby reshaping acceptance pathways under conditions of fluctuating physical and cognitive capacity.

Moderation analyses show that increasing symptom burden is associated with a weaker relationship between perceived ease of use and behavioral intention (*β* = −0.24, *p* = .042), while simultaneously amplifying the inhibitory effect of technological anxiety on intention (*β* = −0.28, *p* = .019). These patterns suggest that identical evaluative states may translate into different behavioral intentions depending on momentary symptom severity. Under higher symptom burden, concerns related to uncertainty, reliability, and perceived risk appear to gain salience, whereas usability considerations recede in importance.

Importantly, symptom severity did not uniformly suppress acceptance-related evaluations. Instead, its effects were selective and asymmetric, reinforcing emotional inhibition while attenuating cognitive facilitation. This supports the interpretation of symptom severity as a situational constraint rather than a stable dispositional barrier, influencing how individuals weigh effort, risk, and anticipated benefit at a given point in time.

Taken together, these findings highlight the limitations of static acceptance models when applied to conditions characterized by fluctuating capability. In MS, acceptance pathways appear to be state-dependent, with symptom dynamics shaping not whether technologies are valued in principle, but whether they are perceived as manageable and actionable in practice. This contextual role of symptom severity provides a direct conceptual bridge between the dominance of emotional determinants discussed in Section 4.2 and the disease-sensitive framework synthesized in Section 4.4.

### 4.4 Toward a conceptual extension of TAM for multiple sclerosis

Based on the present findings, we outline foundational elements of a conceptual, disease-sensitive extension of established technology acceptance frameworks for Multiple Sclerosis (MS) (Figure 1). Importantly, this extension is not proposed as a validated structural model, but as a theoretically informed synthesis of exploratory empirical associations observed in an MS population.

Within this exploratory framework, trust in technology emerged as the most salient predictor of behavioral intention, while technological anxiety acted as a central inhibitory factor whose impact was amplified under higher symptom severity. In contrast, perceived usefulness and perceived ease of use played secondary roles, and their relevance diminished in phases of increased symptom burden. These patterns suggest that emotional and capability-related factors may outweigh cognitive appraisals when users face fluctuating physical and cognitive resources.

Furthermore, the attenuated intention–behavior relationship observed in the MS group highlights a critical limitation of traditional acceptance models, which typically assume a stable translation of intention into use. In MS, capability constraints related to fatigue, cognitive load, and symptom unpredictability appear to disrupt this pathway, indicating that intention alone may be an insufficient proxy for actual adoption in fluctuating neurological conditions.

The proposed conceptual extension therefore integrates three disease-relevant elements into existing TAM/UTAUT structures:

1. Emotional determinants (trust and anxiety) as primary drivers of intention,
2. Symptom severity as a moderator that reshapes acceptance pathways, and
3. A capability-sensitive intention–behavior link that accounts for sporadic or inconsistent use patterns.

Taken together, these elements point toward a precision-oriented understanding of technology acceptance that explicitly considers vulnerability, fluctuating capacity, and emotional risk appraisal. The present study provides initial empirical signals supporting this perspective, while formal model testing and validation require future research using longitudinal designs and structural modeling approaches.

A structured comparison of the proposed Extended D-TAM with traditional TAM and UTAUT frameworks is presented in Supplementary Table S3.

### 4.5 Practical implications for digital health design

Our findings provide an empirical basis for MS-specific design recommendations that directly reflect the observed acceptance patterns. A detailed evidence-to-design translation matrix linking empirical findings to concrete design implications is provided in Supplementary Table S2.

#### 4.5.1 Prioritize trust-building over simplicity optimization

Trust in Technology (*β* = .52, *p* < .001) emerged as the strongest predictor of intention—far outweighing usefulness or ease of use. Effective strategies include: (1) plain-language algorithmic transparency; (2) clinician-in-the-loop features that leverage trust transfer; (3) explicit uncertainty communication; and (4) accessible audit trails. These measures address the central emotional driver of adoption more effectively than further interface simplification.

#### 4.5.2 Reduce anxiety through supportive, low-stakes design

Technological Anxiety strongly inhibited intention (*β* = –.38, *p* < .001), with effects intensifying under high symptom severity. Helpful countermeasures include: (1) low-stakes onboarding with observational modes; (2) proactive reassurance; (3) easy access to human support; and (4) fine-grained privacy controls. These elements create emotional safety in moments of cognitive fatigue or uncertainty.

#### 4.5.3 Accommodate fluctuating capability

Symptom severity moderated the PEOU→BI link, indicating that ease of use becomes less relevant during high-fatigue phases. Systems should adapt to capability states through: (1) cognitive-load–responsive interface simplification; (2) multimodal interaction options; (3) postponement of non-urgent decisions; and (4) context-aware timing aligned with individual energy patterns.

#### 4.5.4 Design for sporadic use patterns

The intention–behavior gap—similar intentions but substantially lower regular use—suggests that fluctuating capability, not motivation, drives disengagement. Design adaptations include: (1) prioritizing passive over active monitoring; (2) tolerance for irregular data input; (3) supportive re-engagement messaging; and (4) maintaining value during inactive periods via ambient sensing.

These recommendations differ from generic digital-health design principles by emphasizing emotional safety and capability accommodation. Effective MS-focused tools must feel forgiving and supportive, not demanding.

### 4.6 Limitations and future directions

The comparison group (*n* = 14) was substantially smaller than the MS group, limiting power for between-group analyses; these findings should therefore be interpreted as exploratory. Although internal consistencies were high, the exploratory factor analysis (EFA) was conducted with a modest sample-to-item ratio and should be viewed as providing only approximate evidence of construct separability. Future studies should validate the structure with confirmatory approaches.

The cross-sectional design does not permit causal inference; longitudinal data are needed to examine how symptom fluctuations affect attitudes and use over time. Actual technology use and symptom severity relied on self-report rather than objective measurement, introducing several potential biases. First, social desirability bias may lead to overreporting of both usage intentions and actual behavior, potentially underestimating the true size of the intention-behavior gap observed in MS. Second, recall bias may differentially affect participants with higher cognitive load, leading to less accurate reporting of sporadic technology interactions. Third, subjective symptom ratings may not capture clinically relevant fluctuations that influence technology use. Future research should employ objective usage logs (e.g., passive wearable tracking, app engagement timestamps) and validated clinical instruments (EDSS) to determine whether the observed intention-behavior gap reflects disease-specific capability constraints or measurement error. If objective measures reveal even lower usage than self-reported, this would strengthen the capability-sensitive framework we propose.

Finally, the study focused on acceptance rather than clinical effectiveness. Whether improved acceptance translates into better health outcomes remains to be tested.

Digital health literacy represents an additional important factor that was not assessed in this study. Recent research suggests that digital health literacy may be particularly critical for vulnerable populations with chronic diseases (27), potentially mediating the relationship between acceptance and actual use. Future studies should incorporate digital health literacy measures alongside traditional acceptance constructs.

Importantly, while we propose foundational elements toward an Extended D-TAM for MS, this model requires formal validation through structural equation modeling with larger samples. Our exploratory regression analyses support the theoretical rationale but do not constitute comprehensive model testing. Future research should examine the integrated structural model, assess fit indices, and conduct nested model comparisons against traditional TAM/UTAUT frameworks.

Finally, generalizability to other fluctuating neurological conditions (e.g., Parkinson's, epilepsy) should be examined—the Extended D-TAM may offer a template beyond MS. Additionally, while our study focused on middle-aged MS patients (mean age 46.8 years), future research should examine whether these acceptance patterns differ in older populations, where additional age-related factors such as reduced digital literacy and increased technological anxiety may further complicate technology adoption (28).

### 4.7 Theoretical and practical contributions

This study provides exploratory evidence suggesting that emotional factors may outweigh cognitive appraisal under vulnerability conditions, that disease-specific moderators fundamentally alter acceptance pathways, and that the intention-behavior gap constitutes a critical phenomenon in chronic illness requiring models that account for capability fluctuations. Practically, findings inform digital health design: prioritizing trust-building over simplicity, reducing anxiety through supportive interactions, accommodating cognitive fluctuations, and designing for sporadic use patterns. These strategies differ fundamentally from generic guidelines by emphasizing emotional safety and capability accommodation over engagement optimization.

## 5 Conclusion

This exploratory study provides a disease-sensitive perspective on digital health acceptance among persons with Multiple Sclerosis. Rather than challenging established acceptance models, the findings suggest that emotional determinants and capability-related constraints reshape how classical acceptance constructs operate under conditions of fluctuating cognitive and physical capacity. Trust in technology and technological anxiety emerged as central drivers of behavioral intention, while perceived usefulness and ease of use appeared attenuated and partially overlapping, particularly under higher symptom burden.

Importantly, symptom severity functioned as a contextual moderator, amplifying emotional inhibition and weakening the translation of intention into actual use. These patterns highlight the limitations of static acceptance models when applied to neurological conditions characterized by variability and uncertainty. The conceptual framework proposed in this study synthesizes these insights into a disease-sensitive perspective on technology acceptance that emphasizes emotional appraisal, momentary capability, and state-dependent intention–behavior relationships.

Future research should build on these findings using longitudinal designs and objective usage measures to further clarify how acceptance dynamics evolve across symptom phases. From a practical perspective, digital health interventions for MS may benefit from designs that reduce perceived effort, address emotional risk appraisal, and accommodate fluctuating user capacity.

## Data Availability

The datasets presented in this study can be found in online repositories. The names of the repository/repositories and accession number(s) can be found below: 10.5281/zenodo.17857319.
