# Supplementary material for: Trust and anxiety as primary drivers of digital health acceptance in multiple sclerosis: toward an extended disease-specific technology acceptance model
Source: Front Digit Health. 2026 Mar 13;8:1763329. doi: 10.3389/fdgth.2026.1763329 (PMC13021651; doi:10.3389/fdgth.2026.1763329)
Supplement: Supplementary file 1 [file Datasheet1.pdf]

SUPPLEMENTARY MATERIALS

Supplementary Table S1: Complete Item List with Factor Loadings

Perceived Usefulness (α = .89)

1. AI-supported health apps would help me better manage my MS symptoms (.82)
2. An AI app that warns me when my body temperature rises too much would be useful (.85)
3. A wearable that monitors my gait pattern could help detect MS relapses early (.78)
4. AI-based symptom prediction would improve my quality of life (.81)
5. Digital health tools could help me make better health decisions (.79)

Perceived Ease of Use (α = .87)

1. Learning to use AI health apps would be easy for me (.84)
2. I would find wearable devices simple to operate (.81)
3. Interacting with AI-supported health tools would not require much mental effort (.76)
4. I could easily integrate digital health tools into my daily routine (.79)
5. Using AI health apps would be clear and understandable (.83)

Behavioral Intention (α = .91)

1. I intend to use AI-supported health apps in the next 6 months (.88)
2. I would recommend wearable health devices to other MS patients (.85)
3. I plan to try digital health tools for symptom monitoring (.89)
4. I would continue using digital health apps if I started (.86)
5. I am willing to share health data with AI systems if it improves my care (.82)

Social Influence (α = .83)

1. My neurologist would support me using digital health tools (.79)
2. Other MS patients I know use wearable devices (.74)
3. People important to me think I should use AI health apps (.81)
4. My family encourages me to try digital health solutions (.77)
5. Healthcare professionals recommend digital monitoring tools (.80)

Trust in Technology (α = .88)

1. I trust that AI-based health apps analyze my health data accurately (.86)
2. I believe wearable devices provide reliable health measurements (.84)
3. I trust that my health data is kept secure by digital health providers (.82)

4. I have confidence that AI algorithms can detect meaningful health patterns (.85)
5. I trust that digital health tools would alert me appropriately to concerning symptoms (.81)

Technological Anxiety (α = .85)

1. I worry that AI health apps might give me incorrect information (R) (.80)
2. Using complex digital health tools makes me nervous (R) (.83)
3. I fear that I might not interpret data from wearables correctly (R) (.78)
4. I am concerned that AI systems might miss important health changes (R) (.82)
5. Technology-related tasks in health management make me anxious (R) (.79)

Note. R = reverse coded. Factor loadings from principal axis factoring with oblimin rotation.

Supplementary Table S2: Evidence-to-Design Translation Matrix

**Empirical Finding**	**Statistical Evidence**	**Design Implication**	**Mechanism**
TT strongest predictor	$\beta = .52, p < .001$	Algorithmic transparency, clinician integration	Builds primary adoption driver
TA strong inhibitor	$\beta = -.38, p < .001$	Reassuring communication, escape routes	Reduces primary barrier
Symptom severity × TA	$\beta = -.28, p = .019$	Adaptive anxiety reduction in high-symptom states	Prevents anxiety spiral
Symptom severity × PEOU	$\beta = -.24, p = .042$	Cognitive load adaptation, not just simplification	Accommodates capability fluctuation
Intention-behavior gap	$\chi^2 = 7.83, p = .020$	Design for sporadic use, passive monitoring	Addresses capability constraints
Moderate SI effects	$\beta = .23, p = .021$	Clinical integration, prescription framing	Leverages trust transfer
PU/PEOU weak predictors	$\beta = .18/.14$, n.s.	Deprioritize feature richness over emotional safety	Aligns with actual decision drivers

Supplementary Table S3: Comparison of Extended D-TAM for MS with Traditional Models

**Aspect**	**TAM (Davis, 1989)**	**UTAUT (Venkatesh et al., 2003)**	**Extended D-TAM for MS**
**Primary drivers**	PU, PEOU	Performance Expectancy, Effort Expectancy	TT, TA (with PU, PEOU secondary)
**Emotional factors**	Not included	Limited (facilitating conditions)	Central (TT, TA as main predictors)

Aspect	TAM (Davis, 1989)	UTAUT (Venkatesh et al., 2003)	Extended D-TAM for MS
**Disease-specific moderators**	None	Generic (age, gender, experience)	Symptom severity, cognitive load, fatigue
**Intention-behavior link**	Strong, direct	Strong, direct	Attenuated, mediated by capability
**Target population**	General users	General users	MS patients with fluctuating symptoms
**Explained variance (BI)**	~40% typical	~50% typical	61% in this study
